# Race and Ethnicity, Gender, and Promotion of Physicians in Academic Medicine

**DOI:** 10.1001/jamanetworkopen.2024.46018

**Published:** 2024-11-27

**Authors:** Lauren Clark, Elena Shergina, Nathalia Machado, Taneisha S. Scheuermann, Nasrin Sultana, Deepika Polineni, Grace H. Shih, Robert D. Simari, Jo A. Wick, Kimber P. Richter

**Affiliations:** 1 Department of Biostatistics and Data Science, University of Kansas School of Medicine, Kansas City; 2 Adult and Child Center for Outcomes Research and Delivery Science, University of Colorado School of Medicine, Aurora; 3 Department of Population Health, University of Kansas School of Medicine, Kansas City; 4 Department of Pediatrics, Division of Allergy and Pulmonary Medicine, Washington University School of Medicine, St Louis, Missouri; 5 Department of Anesthesiology, University of Kansas School of Medicine, Kansas City; 6 Department of Cardiovascular Medicine, University of Kansas School of Medicine, Kansas City

## Abstract

**Importance:**

The ranks of academic physicians do not reflect the diversity of the US population. To create a diverse and effective medical workforce, it is important to know the extent to which gender, race and ethnicity, and the intersection of these factors are associated with career advancement.

**Objective:**

To assess whether race and ethnicity and gender are associated with appointment to or promotion within academic medicine.

**Design, Setting, and Participants:**

This cohort study used Association of American Medical Colleges data on graduates of all US MD-granting medical schools from 1979 to 2019 merged with faculty appointment data since 2000. Data for this study are based on a February 19, 2021, snapshot. The analysis was performed from March 8, 2021, to May 5, 2023.

**Exposure:**

Gender and race and ethnicity among physician graduates.

**Main Outcomes and Measures:**

The main outcome was full-time faculty appointments and promotions to the ranks of instructor, assistant professor, associate professor, full professor, and department chair. Cox proportional hazards models were used to examine the differences in likelihood of appointment and promotion for racial and ethnic minority men and women compared with White men.

**Results:**

The analytic sample included 673 573 graduates (mean [SD] age at graduation, 28.1 [3.2] years; 59.7% male; and 15.2% identifying as Asian, 6.1% as Black, and 69.6% as White). White men accounted for the largest subgroup of graduates (43.8%). Asian men, Asian women, Black women, and White women were more likely than White men to be appointed to entry-level positions. Among physicians graduating both before and after 2000, White men were more likely to be promoted to upper ranks compared with physicians of nearly every other combination of gender and race and ethnicity. Among physicians graduating prior to 2000, Black women were 55% less likely (HR, 0.45; 95% CI, 0.41-0.49) to be promoted to associate professor (n = 131 457) and 41% less likely (HR, 0.59; 95% CI, 0.51-0.69) to be promoted to full professor (n = 43 677) compared with White men. Conversely, Black men (HR, 1.29; 95% CI, 1.03-1.61) were more likely to be appointed as department chair (n = 140 052) than White men.

**Conclusions and Relevance:**

These findings indicate that preferential promotion of White men within academic medicine continues to persist in the new millennium, with racially and ethnically diverse women experiencing greater underpromotion. To achieve a workforce that reflects the diversity of the US population, this study suggests that academic medicine needs to transform its culture and practices surrounding faculty appointments and promotions.

## Key Points

**Question:**

What is the association of gender and race and ethnicity with advancement in academic medicine?

**Findings:**

In this cohort study of 673 573 physician-graduates from 1979 to 2019, Asian men, Asian women, Black women, and White women were more likely than White men to be appointed to entry-level academic medicine positions. Black women graduating prior to 2000 were 55% less likely to be promoted to associate professor and 41% less likely to be promoted to full professor than White men.

**Meaning:**

These findings suggest that compared with White men, women and racial and ethnic minority groups are more likely to enter academic medicine but, with few exceptions, are less likely to achieve promotion to upper ranks.

## Teaser

This cohort study examines the association of race and ethnicity and gender with appointment to or promotion within academic medicine in the US over the past 40 years.

## Introduction

Medical school faculty training the next generation of physicians do not resemble the diversity of America. Compared with the US working population, American Indian, Black, and Hispanic people are underrepresented in the physician workforce.^1^ Pipeline programs may not change matters as these groups are also underrepresented among trainees in medical schools compared with the US census.^2^ Female physicians in US medical schools are less likely than male physicians to be promoted to associate or full professor or appointed to department chair, with no observed narrowing in these gaps over a 35-year period.^3^ Physicians identifying with racial and ethnic minority groups that are underrepresented in medicine are promoted at lower rates than their Asian and White peers,^4^ and the proportion of faculty from these groups has remained the same or worsened over time.^5^ Although numerous studies examined the intersectional association of race and ethnicity and gender with physical health,^6^ none examined whether this overlap is associated with career advancement in academic medicine. Differential rates of promotion may partially account for disparities between the gender and racial and ethnic makeup of the US population and medical school faculty.

Patients have been observed to fare better when treated by physicians with a similar background to theirs. Poor health has been disproportionately associated with vulnerable groups, including racial and ethnic minority groups.^7^ Gender and racial and ethnic concordance between patients and physicians is associated with higher patient satisfaction, likelihood to visit a physician, and receipt of preventive services.^8,9,10^ Notably, Black newborns are more than twice as likely to die in their first year compared with White newborns, but the mortality gap is smaller when Black physicians deliver care for Black newborns compared with White physicians.^11^ Because the US is rapidly becoming more culturally and racially diverse,^12,13^ ensuring diversity in the health care profession may help reduce inequalities in medical care.^14^

It is important to know whether diversity in race and ethnicity, gender, and the intersection of these factors, which could strengthen our nation’s ability to deliver high-quality care, is associated with academic physicians achieving career advancement. Following promotion, workers may experience more job security, more decision-making freedom, more fairness in pay and higher job satisfaction.^15^ Unsurprisingly, poor career advancement and promotion have been cited as the top causes of leaving academic medicine.^16,17^ This attrition undermines the ability of academic health centers to fulfill their missions of discovery, education, and patient care.^18^

Developing diversity in faculty leadership may help improve the health of the US population, which has a shorter life expectancy and worse health outcomes compared with other countries with advanced economies.^12^ Students at medical schools with more diverse faculty have reported being better prepared to care for minority populations,^19^ which may reduce health care disparities. The purpose of our study was to pool 41 cohorts of MD graduates to determine the association of the intersection of gender and race and ethnicity with promotion of physicians in academic medicine. We examined whether more recent cohorts of graduates are closing the gap compared with those who graduated prior to 2000.

## Methods

### Data Sources and Study Sample

This cohort study used data from the Association of American Medical Colleges (AAMC) Student Records System, which includes every graduate of US MD-granting medical schools. Data from the AAMC Faculty Roster include information on every full-time faculty or department chair appointment at a US MD-granting institution ever held by a US MD graduate. The roster data for this study are based on a February 19, 2021, snapshot. As this study involved analysis of existing, deidentified data, ethics approval and informed consent were not required by the University of Kansas Medical Center Institutional Review Board. The study adheres to the Strengthening the Reporting of Observational Studies in Epidemiology (STROBE) reporting guideline.

### Study Measures

Our main outcome measures were full-time faculty appointments and promotions at the level of instructor, assistant professor, associate professor, full professor, and department chair. Six appointments and promotions were examined for the analysis, and all were considered separately. A rank advancement was considered an appointment or promotion regardless of whether it was an internal advancement or a move to another institution. We refer to advancements to the ranks of instructor and chair as appointments because entry into these ranks typically occurs outside of the promotion and tenure process; this is also true for the rank of assistant professor when it serves as the entry rank for graduates. We refer to advancements to the ranks of assistant professor following instructor, associate professor, and full professor as promotions because these typically occur as a result of the promotion and tenure process. Data on specific types of faculty tracks (clinician, educator, and researcher) were not available. We also did not have information about tenure status or academic productivity, such as research publications. Data on ranks included medical school (deidentified; code number only), department type, and start and end dates. Available demographic data on graduates were graduation year and self-reported gender and race and ethnicity.

### Statistical Analysis

The data analysis was performed from March 8, 2021, to May 5, 2023. Race and ethnicity were provided as a combined variable. Prior to academic year 2002-2003, only 1 race and ethnicity category could be chosen. From academic years 2002-2003 until 2012-2013, race and ethnicity were asked in 2 separate questions. From academic year 2013-2014 to present, race and ethnicity were collected in 1 question in which any combination of races and ethnicities could be chosen.^20^ To standardize these collection differences, race and ethnicity were classified into 6 distinct categories: American Indian, Alaska Native, Native Hawaiian or Pacific Islander, non-Hispanic; Asian, non-Hispanic only; Black, non-Hispanic only; Hispanic, Latino, of Spanish origin, or multiracial Hispanic; White, non-Hispanic only; and other, which included individuals who self-reported their race and ethnicity as other, non-Hispanic multiracial, or unknown.

We used Cox proportional hazards models to examine differences between racial and ethnic minority women, White women, and racial and ethnic minority men compared with White men. The models accounted for the interaction between gender and race and ethnicity, race and ethnicity and graduation cohort, and gender and graduation cohort. Graduation cohort was determined based on the year of graduation, with graduation dates before 2000 in the earlier cohort and graduation dates after 2000 in the later cohort.

Survival analysis is a statistical method particularly suited for studying time-to-event data, such as faculty promotions or appointments in academic medicine. A key advantage of this statistical approach is its ability to handle censored data, which is a unique type of missing data that occurs when an individual is not appointed or promoted during the study period. Survival analysis formally incorporates the time individuals are eligible for appointment or promotion, regardless of censoring, providing a more accurate estimate of the event rate. In this study, individuals were considered eligible for a promotion or an appointment starting on the first day of their previous academic rank. For example, individuals seeking an associate professor position were considered eligible from the date they were appointed an assistant professor. The only exception was for appointments to instructor or assistant professor after graduation, which used June 30 of their graduation year as the date they were considered eligible for appointment. We used right censoring to account for individuals who were not promoted or appointed during the study period. Specifically, individuals who left or took a lengthy hiatus from academic medicine, which was defined as a gap in subsequent appointments of 3 or more years, were censored at 3 years after the end date of their most recent appointment. Faculty without a promotion by February 19, 2021, were censored on that date. Finally, to minimize the impact of bias that could result from differences in the length of observation in early and later cohorts, we censored data for all individuals, regardless of cohort, at the maximum follow-up time observed in the later cohort (2000-2019). Data were censored at 7539 days for appointment to instructor after graduation, at 7539 days for appointment to assistant professor after graduation, 7538 days for promotion to assistant professor after instructor, 7539 days for promotion to associate professor after assistant professor, 6575 days for promotion to full professor after associate professor, and 7539 days for appointment to department chair after initial appointment in academic medicine at any level. The analyses were conducted using SAS, version 9.4.23 (SAS Institute Inc).

## Results

### Initial Appointments in Academic Medicine

Data were merged into a single file containing information on 693 965 persons who graduated from US MD-granting medical schools in academic years 1978-1979 through 2019-2020. After excluding 3 individuals due to unknown gender and 20 389 due to a graduation date after 2019, our sample consisted of 673 573 graduates (mean [SD] age at graduation, 28.1 [3.2] years; 40.3% female; 59.7% male; and 0.4% identifying as American Indian, Alaska Native, Native Hawaiian, or Pacific Islander; 15.2% as Asian; 6.1% as Black; 6.2% as Hispanic; 69.6% as White; and 2.4% as other race and ethnicity).

**Figure 1.**
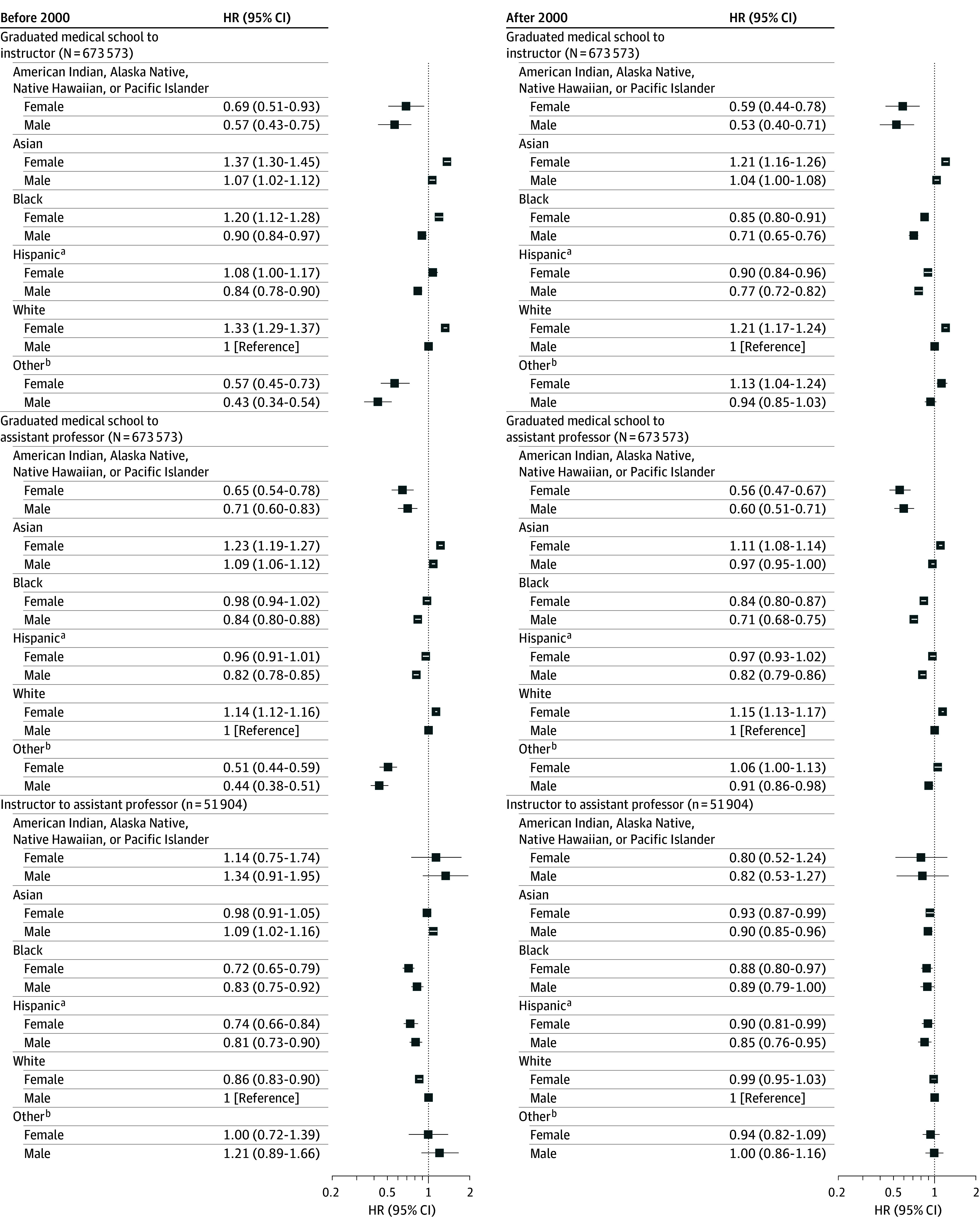
Likelihood of Appointments to Positions of Instructor and Assistant Professor and Promotion From Instructor to Assistant Professor Among Female and Male Physicians by Race and Ethnicity HR indicates hazard ratio. ^a^Hispanic, Latino, of Spanish origin, or multiracial Hispanic. ^b^Other, multiracial non-Hispanic, or unknown.

White men did not dominate initial appointments at the instructor or the assistant professor levels. Both before and/or after 2000, Asian (hazard ratio [HR], 1.37 [95% CI, 1.30-1.45] and 1.21 [95% CI, 1.16-1.26]), Black (before 2000: HR, 1.20 [95% CI, 1.12-1.28]), and White (HR, 1.33 [95% CI, 1.29-1.37] and 1.21 [95% CI, 1.17-1.24]) women were more likely to enter academia at the instructor level compared with White men in the same cohort (Figure 1; eTable 1 in Supplement 1). Hazard ratios across gender, race and ethnicity, and graduation cohort were significant except for Hispanic women graduating before 2000 and men of other race and ethnicity graduating after 2000. Before and/or after 2000, Asian men (before 2000: HR, 1.09 [95% CI, 1.06-1.12]), Asian women (HR, 1.23 [95% CI, 1.19-1.27] and 1.11 [95% CI, 1.08-1.14]), White women (HR, 1.14 [95% CI, 1.12-1.16]) and 1.15 [95% CI, 1.13-1.17]), and women of other race and ethnicity (after 2000: HR, 1.06 [95% CI, 1.00-1.13]) were more likely to enter academia at the assistant professor level compared with White men (Figure 1; eTable 2 in Supplement 1). Among instructors, prior to 2000, Asian men had a higher likelihood of promotion to assistant professor (HR, 1.09; 95% CI, 1.02-1.16), but after 2000, no groups outperformed White men (Figure 1; eTable 3 in Supplement 1).

### Promotion to Higher Ranks

**Figure 2.**
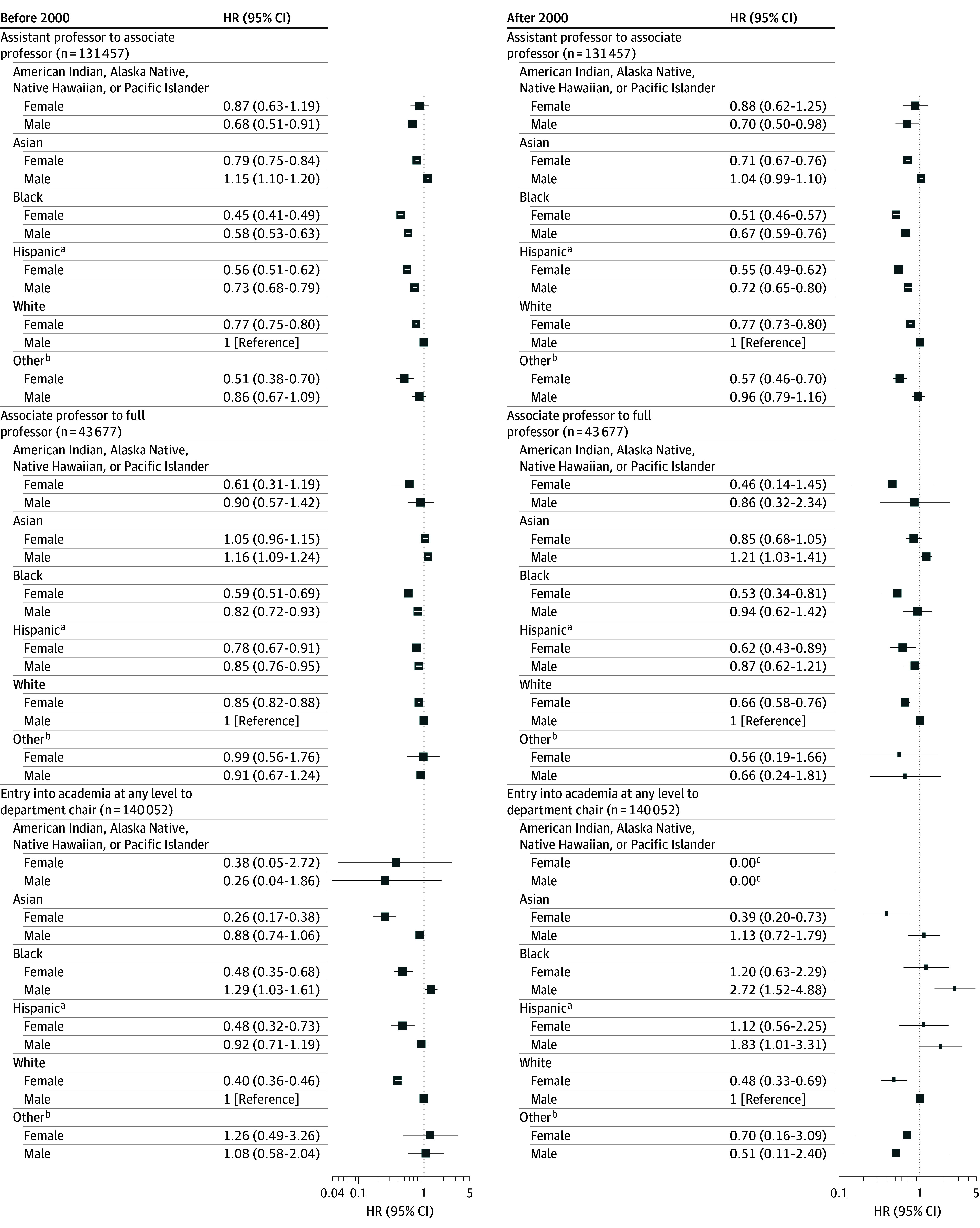
Likelihood of Promotion to Upper Ranks in Academic Medicine Among Female and Male Physicians by Race and Ethnicity A different scale was used due to small sample size. HR indicates hazard ratio. ^a^Includes Hispanic, Latino, Spanish origin, or multiracial Hispanic. ^b^Includes other, multiracial non-Hispanic, or unknown. ^c^The 95% CI overlapped the reference category (female: 95% CI, 0.00-1.78 × 10^63^; male: 95% CI, 0.00-1.03 × 10^63^).

For promotion from assistant professor to associate professor, 131 457 assistant professors were included in the analysis, and the majority were White men (44.0%). All racial and ethnic groups of both female and male gender had a lower likelihood of promotion compared with White men, with the exception of Asian men (Figure 2). Only 50 of 173 (28.9%) women of American Indian, Alaska Native, Native Hawaiian, or Pacific Islander heritage were promoted, and their HR of promotion compared with White men was not significant (Table 1). Prior to 2000, Asian women were 21% less likely (HR, 0.79; 95% CI, 0.75-0.84), Black women 55% less likely (HR, 0.45; 95% CI, 0.41-0.49), Hispanic women 44% less likely (HR, 0.56; 95% CI, 0.51-0.62), and White women 23% less likely (HR, 0.77; 95% CI, 0.75-0.80) to be promoted compared with White men (Figure 2; Table 1). Comparison of early cohorts with later cohorts revealed that lower likelihoods of promotion compared with White men did not change markedly (Table 1).

**Table 1.** Likelihood of Promotion From Assistant Professor to Associate Professor (N = 131 457)^a^

Group	No. of physicians (column %)	Events, No. (%)	Graduation date, HR (95% CI)	
			Before 2000	After 2000
No. (row %)	NA	NA	68 390 (52.0)	63 067 (48.0)
Events, No. (%)	NA	NA	26 359 (38.5)	10 637 (16.9)
American Indian, Alaska Native, Native Hawaiian, or Pacific Islander				
Female	173 (0.1)	50 (28.9)	0.87 (0.63-1.19)	0.88 (0.62-1.25)
Male	236 (0.2)	63 (26.7)	0.68 (0.51-0.91)	0.70 (0.50-0.98)
Asian				
Female	9340 (7.1)	1844 (19.7)	0.79 (0.75-0.84)	0.71 (0.67-0.76)
Male	10 519 (8.0)	3147 (29.9)	1.15 (1.10-1.20)	1.04 (0.99-1.10)
Black				
Female	4204 (3.2)	688 (16.4)	0.45 (0.41-0.49)	0.51 (0.46-0.57)
Male	2713 (2.1)	604 (22.3)	0.58 (0.53-0.63)	0.67 (0.59-0.76)
Hispanic^b^				
Female	3167 (2.4)	525 (16.6)	0.56 (0.51-0.62)	0.55 (0.49-0.62)
Male	3658 (2.8)	824 (22.5)	0.73 (0.68-0.79)	0.72 (0.65-0.80)
White				
Female	37 479 (28.5)	9422 (25.1)	0.77 (0.75-0.80)	0.77 (0.73-0.80)
Male	57 860 (44.0)	19 585 (33.8)	1 [Reference]	1 [Reference]
Other^c^				
Female	1074 (0.8)	88 (8.2)	0.51 (0.38-0.70)	0.57 (0.46-0.70)
Male	1034 (0.8)	156 (15.1)	0.86 (0.67-1.09)	0.96 (0.79-1.16)

Abbreviations: HR, hazard ratio; NA, not applicable.

^a^ Of the original sample of 673 573, 541 742 individuals were excluded because they never held an assistant professor appointment, and 374 individuals were excluded because they were promoted to associate professor before being appointed to assistant professor. Of the 131 457 assistant professors included in the analysis, 36 996 were promoted to associate professor (events), and 94 461 were censored: 271 were later promoted to associate professor but were censored because they reached the maximum allowed time before associate professor promotion (7539 days), 41 993 were censored on the last date of file, and 52 197 were censored on last date of current assistant professor promotion plus 3 years.

^b^ Includes Hispanic, Latino, of Spanish origin, or multiracial Hispanic.

^c^ Includes other, multiracial non-Hispanic, or unknown.

For promotion from associate professor to full professor, 43 677 associate professors were included in the analysis, and the majority were White men (53.9%). All racial and ethnic groups of both female and male genders had a lower likelihood of promotion compared with White men, with the exception of Asian men graduating both before and after 2000 (HR, 1.16 [95% CI, 1.09-1.24] and 1.21 [95% CI, 1.03-1.41]) (Figure 2). Only 9 of 53 women of American Indian, Alaska Native, Native Hawaiian, or Pacific Islander heritage were promoted, and their HR of promotion compared with White men was not significant (Table 2). Prior to 2000, Asian women were 5% more likely (HR, 1.05; 95% CI, 0.96-1.15), Black women 41% less likely (HR, 0.59; 95% CI, 0.51-0.69), Hispanic women 22% less likely (HR, 0.78; 95% CI, 0.67-0.91), and White women 15% less likely (HR, 0.85; 95% CI, 0.82-0.88) to be promoted compared with White men (Table 2). Comparison of early cohorts with later cohorts revealed that disparities compared with White men did not change markedly with the exception of White women, who experienced a decrease in likelihood of promotion to full professor in the early cohort (HR, 0.85; 95% CI, 0.82-0.88) and a drop in the later cohort (HR, 0.66; 95% CI, 0.58-0.76) (Table 2).

**Table 2.** Likelihood of Promotion From Associate Professor to Full Professor (N = 43 677)^a^

Group	No. of physicians (column %)	Events, No. (%)	Graduation date, HR (95% CI)	
			Before 2000	After 2000
No. (row %)	NA	NA	32 306 (74.0)	11 371 (26.0)
Events, No. (%)	NA	NA	15 086 (46.7)	961 (8.5)
American Indian, Alaska Native, Native Hawaiian, or Pacific Islander				
Female	53 (0.1)	9 (17.0)	0.61 (0.31-1.19)	0.46 (0.14-1.45)
Male	77 (0.2)	21 (27.3)	0.90 (0.57-1.42)	0.86 (0.32-2.34)
Asian				
Female	2037 (4.7)	534 (26.2)	1.05 (0.96-1.15)	0.85 (0.68-1.05)
Male	3571 (8.2)	1199 (33.6)	1.16 (1.09-1.24)	1.21 (1.03-1.41)
Black				
Female	804 (1.8)	171 (21.3)	0.59 (0.51-0.69)	0.53 (0.34-0.81)
Male	736 (1.7)	243 (33.0)	0.82 (0.72-0.93)	0.94 (0.62-1.42)
Hispanic^b^				
Female	632 (1.5)	170 (26.9)	0.78 (0.67-0.91)	0.62 (0.43-0.89)
Male	1016 (2.3)	328 (32.3)	0.85 (0.76-0.95)	0.87 (0.62-1.21)
White				
Female	10 938 (25.0)	3510 (32.1)	0.85 (0.82-0.88)	0.66 (0.58-0.76)
Male	23 526 (53.9)	9808 (41.7)	1 [Reference]	1 [Reference]
Other^c^				
Female	99 (0.2)	12 (12.1)	0.99 (0.56-1.76)	0.56 (0.19-1.66)
Male	188 (0.4)	42 (22.3)	0.91 (0.67-1.24)	0.66 (0.24-1.81)

Abbreviations: HR, hazard ratio; NA, not applicable.

^a^ Of the original sample of 673 573, 629 840 individuals were excluded because they never held an associate professor promotion, and 56 individuals were excluded because they were appointed to full professor before being promoted to associate professor. Of the 43 677 associate professors included in the analysis, 16 047 were promoted to full professor (events), and 27 630 were censored: 362 were later promoted to full professor but were censored because they reached the maximum allowed time before full professor promotion (6575 days), 16 634 were censored on the last date of file, and 10 634 were censored on the last date of current associate professor promotion plus 3 years.

^b^ Includes Hispanic, Latino, of Spanish origin, or multiracial Hispanic.

^c^ Includes other, multiracial non-Hispanic, or unknown.

### Appointment to Department Chair

For appointment to department chair, 140 052 faculty were included in the analysis, and the majority were White men (45.0%). White men had a higher likelihood of appointment compared with most other racial and ethnic groups of both female and male gender (Figure 2). Across cohorts, the proportion of White men promoted to department chair (1627 of 294 880 [0.55%]) was similar to the proportion of Black men promoted to chair (85 or 17 360 [0.49%]). Only 1 of 177 (0.6%) women of American Indian, Alaska Native, Native Hawaiian, or Pacific Islander heritage was appointed, and her 95% CIs for the HR of appointment overlapped the reference category of White men (Table 3). Prior to 2000, Asian women were 74% less likely (HR, 0.26; 95% CI, 0.17-0.38), Black women 52% less likely (HR, 0.48; 95% CI, 0.35-0.68), Hispanic women 52% less likely (HR, 0.48; 95% CI, 0.32-0.73), and White women 60% less likely (HR, 0.40; 95% CI, 0.36-0.46) to be appointed compared with White men (Table 3). After 2000, although several racial and ethnic groups of both female and male genders experienced a higher likelihood of being appointed to department chair, only Black men (HR, 2.72; 95% CI, 1.52-4.88) and Hispanic men (HR, 1.83; 95% CI, 1.01-3.31) had significantly higher likelihoods compared with White men (Table 3).

**Table 3.** Entry Into Academic Medicine at Any Level to Department Chair (N = 140 052)^a^

Group	No. of physicians (column %)	Events, No. (%)	Graduation date, HR (95% CI)	
			Before 2000	After 2000
No. (row %)	NA	NA	75 692 (54.1)	64 360 (46.0)
Events, No. (%)	NA	NA	2208 (2.9)	141 (0.1)
American Indian, Alaska Native, Native Hawaiian, or Pacific Islander				
Female	177 (0.1)	1 (0.6)	0.38 (0.05-2.72)	0.00^b^
Male	256 (0.2)	1 (0.4)	0.26 (0.04-1.86)	0.00^b^
Asian				
Female	9626 (6.9)	28 (0.3)	0.26 (0.17-0.38)	0.39 (0.20-0.73)
Male	11 081 (7.9)	144 (1.3)	0.88 (0.74-1.06)	1.13 (0.72-1.79)
Black				
Female	4348 (3.1)	39 (0.9)	0.48 (0.35-0.68)	1.20 (0.63-2.29)
Male	2871 (2.1)	85 (3.0)	1.29 (1.03-1.61)	2.72 (1.52-4.88)
Hispanic^c^				
Female	3293 (2.4)	25 (0.8)	0.48 (0.32-0.73)	1.12 (0.56-2.25)
Male	3897 (2.8)	67 (1.7)	0.92 (0.71-1.19)	1.83 (1.01-3.31)
White				
Female	39 361 (28.1)	317 (0.8)	0.40 (0.36-0.46)	0.48 (0.33-0.69)
Male	62 958 (45.0)	1627 (2.6)	1 [Reference]	1 [Reference]
Other^d^				
Female	1100 (0.8)	5 (0.5)	1.26 (0.49-3.26)	0.70 (0.16-3.09)
Male	1084 (0.8)	10 (0.9)	1.08 (0.58-2.04)	0.51 (0.11-2.40)

Abbreviations: HR, hazard ratio; NA, not applicable.

^a^ Of the original sample of 673 573, 533 024 individuals were excluded because they never entered academic medicine full time at any level, and 497 individuals were excluded because they were appointed to department chair before holding their first full-time faculty position in this dataset. Of the 140 052 faculty members included in the analysis, 2349 were appointed to department chair (events), and 137 703 were censored: 384 were later appointed to chair but were censored because they reached the maximum allowed time before chair appointment (7539 days), 43 897 were censored on last date of the file, and 93 422 were censored on last date of their last faculty appointment plus 3 years.

^b^ The 95% CI overlapped the reference category (female: 95% CI, 0.00-1.78 × 10^63^; male: 95% CI, 0.00-1.03 × 10^63^).

^c^ Includes Hispanic, Latino, of Spanish origin, or multiracial Hispanic.

^d^ Includes other, multiracial non-Hispanic, or unknown.

### Overview of Advancement by Race and Ethnicity and Gender

eTable 4 in Supplement 1 shows that within racial and ethnic categories, graduates of American Indian, Alaska Native, Native Hawaiian, or Pacific Islander heritage were less likely to be appointed to instructor and assistant professor and, in general, had a lower likelihood of being promoted to upper ranks; however, their HRs were not significant. Across all ranks and time frames, compared with White men, Asian men had a higher or equivalent likelihood for promotion, and Asian women had a higher likelihood for appointment or promotion at entry-level ranks only. Black men, who in general had a lower likelihood for promotion at most ranks, had a higher likelihood for appointment to the position of department chair in both the earlier and later cohorts. Hispanic women and men had lower likelihoods of appointment and promotion at most ranks and time frames. Men and women identifying as other race and ethnicity in general had a lower likelihood of promotion across all ranks and time frames, although many HRs were not significant. Compared with White men, White women had a consistently higher likelihood for appointment to entry-level positions. Except for promotion from instructor to assistant professor after 2000, White women had a lower likelihood of promotion for every other rank and time frame.

The eFigure in Supplement 1 compares promotions and appointments between the graduation cohorts before and after 2000. With few exceptions, promotion patterns remained similar for both cohorts.

## Discussion

In this cohort study, Asian female, Asian male, Black female, and White female graduates were more likely than White male graduates to be appointed to entry-level positions in academic medicine. White male graduates prior to 2000 were more likely to be promoted to upper ranks compared with both female and male gender in nearly every other racial and ethnic group. These trends continued in the decades following the year 2000, with few exceptions.

Compared with White men, Black women had the lowest and Hispanic women the second-lowest likelihood of being promoted to both associate and full professor, which held true for cohorts that graduated both before and after 2000. Black and Hispanic women’s likelihoods of promotion were also consistently lower than those of Asian and White women. American Indian, Alaska Native, Native Hawaiian, and Pacific Islander women also had lower likelihoods of being promoted compared with White men at these ranks across both early and late cohorts, but because of small numbers of graduates, the results were not significant.

As medical schools have been recruiting diverse faculty into the field of academic medicine, White male graduates in both the early and late graduate cohorts did not have consistently higher rates of appointment to instructor and assistant professor. However, White men were more likely to be promoted to assistant professor among faculty who entered academic medicine as instructors and had higher likelihoods of promotion to upper ranks and appointment to department chair.

Black men were more likely to be appointed to department chair compared with White men, with the likelihood increasing between early and late cohorts. In the late cohort, Hispanic men also had an increased likelihood of appointment to department chair. Across all cohorts, however, the proportion of White male graduates promoted to department chair (0.55%) was similar to the proportion of Black male graduates promoted to chair (0.49%). Their pathways to department chair differed greatly. Compared with White men, Black men were less likely to enter academic medicine and less likely to be promoted to almost every rank along the way.

### Implications

Medical school faculty do not reflect the diversity in the US population. In our study, White men made up 44.0% and 53.9% of faculty who ever held the position of assistant professor and associate professor, respectively. These percentages are in contrast to non-Hispanic White male representation in the US population of approximately 30%.^21^ Similarly, only 6.6% of 2023 US medical school graduates were Black. Black Americans, however, make up 13.6% of the US population.^21,22^ Although Black male and female graduates had higher likelihoods of being appointed to department chair in the decades since 2000 compared with earlier decades, the lack of representation among medical school graduates suggests that even if women and racial and ethnic minority groups achieve parity in promotion, lack of representation in upper ranks may continue to persist.

This study confirms the findings of other studies that racial and ethnic minority physicians are promoted at lower rates than their White and Asian peers and that these trends are not improving over time.^4,5^ Our study extends these findings by showing that women experience a twofold risk: Racial and ethnic minority female physicians have a lower likelihood of promotion and/or appointment than their male peers. For promotion and appointment, these findings indicate that efforts to ensure equitable advancement should take into consideration how bias might be introduced, and how it might be addressed, at each level of review. Promotions and appointments to leadership positions draw on faculty reviews, which are often conducted by chairs or committees at the department level through a unilateral and/or top-down process. Innovations that may facilitate development include adopting 360-degree performance reviews and incorporating faculty development leadership into the evaluation process.^23^ Promotion of faculty members is recommended by faculty-composed committees based on published criteria for promotion. Conversely, both initial appointments and appointments to chair are typically made by chairs of departments or deans. While both appointment and promotion determinations are made on the basis of performance, notably publications and grants, the disparities in advancement persist when adjusting for these factors.^24,25^ To reduce these disparities, it may be necessary to effectively address racial and ethnic and gender bias in systems of advancement at academic medical centers.^26,27^

### Strengths and Limitations

This study included a large and comprehensive sample built upon previous work^3^ by including the transitions from graduation to instructor and instructor to assistant professor, providing a fuller picture of the academic medicine pipeline. However, our study has several limitations. The dataset only includes full-time faculty appointments; thus, we were not able to differentiate between faculty moved to a part-time or volunteer position and those without any faculty position. We were not able to adjust for research productivity (eg, publications, grants) and the preference of physicians to pursue an academic career, which are both associated with the likelihood of promotion.^28,29^ Since academic year 2013-2014, AAMC has collected finer-tuned categories of race and ethnicity.^20^ These same data do not exist for earlier graduates, which limited our ability to identify trends for important subgroups, such as communities classified as Asian that might be considered underrepresented in medicine.

## Conclusions

In this cohort study, we show that in academic medicine, racial and ethnic minority male physicians were less likely to achieve promotion compared with their White male counterparts. Racial and ethnic minority women experienced a twofold challenge of underpromotion compared with their male counterparts and White men. These associations have not changed in recent years, and prior studies suggest that these difference persist even when adjusting for productivity, such as publications and grants.^28,29^ To achieve a workforce that reflects the diversity of the US population, academic medicine must transform its culture and the practices that surround faculty appointments and promotions.

## Notes

Supplement 1. **eTable 1.** Graduated Medical School to Instructor **eTable 2.** Graduated Medical School to Assistant Professor **eTable 3.** Instructor to Assistant Professor **eTable 4.** Summary of Appointments and Promotions **eFigure.** Summary of Appointments and Promotions Before and After 2000

Supplement 2.
Data Sharing Statement
