# Supplementary material for: Race and Ethnicity, Gender, and Promotion of Physicians in Academic Medicine
Source: JAMA Netw Open. 2024 Nov 27;7(11):e2446018. doi: 10.1001/jamanetworkopen.2024.46018 (PMC12262150; doi:10.1001/jamanetworkopen.2024.46018)
Supplement: Supplement 1. — eTable 1. Graduated Medical School to Instructor eTable 2. Graduated Medical School to Assistant Professor eTable 3. Instructor to Assistant Professor eTable 4. Summary of Appointments and Promotions eFigure. Summary of Appointments and Promotions Before and After 2000 [file jamanetwopen-e2446018-s001.pdf]

Supplemental Online Content

Clark L, Shergina E, Machado N, et al. Race and ethnicity, gender, and promotion of physicians in academic medicine. *JAMA Netw Open*. 2024;7(11):e2446018. doi:10.1001/jamanetworkopen.2024.46018

eTable 1. Graduated Medical School to Instructor

eTable 2. Graduated Medical School to Assistant Professor

eTable 3. Instructor to Assistant Professor

eTable 4. Summary of Appointments and Promotions

eFigure. Summary of Appointments and Promotions Before and After 2000

This supplemental material has been provided by the authors to give readers additional information about their work.

© 2024 Clark L et al. *JAMA Network Open.*

eTable 1. Graduated Medical School to Instructor

Race	Gender				Graduation Date	
					Before 2000	After 2000
				N (Row %)	330,396 (49.1%)	343,177 (51.0%)
				Events (% of N)	24,959 (7.6%)	27,099 (7.9%)
		N (Column %)	Events (% of N)	HR* (95% CI)		
American Indian, Alaska Native, Native Hawaiian, or Pacific Islander	Female	1,260 (0.2%)	65 (5.2%)		0.69 (0.51, 0.93)	0.59 (0.44, 0.78)
	Male	1,620 (0.2%)	72 (4.4%)		0.57 (0.43, 0.75)	0.53 (0.40, 0.71)
Asian	Female	46,326 (6.9%)	4,125 (8.9%)		1.37 (1.30, 1.45)	1.21 (1.16, 1.26)
	Male	55,915 (8.3%)	4,300 (7.7%)		1.07 (1.02, 1.12)	1.04 (1.00, 1.08)
Black	Female	23,896 (3.6%)	1,757 (7.4%)		1.20 (1.12, 1.28)	0.85 (0.80, 0.91)
	Male	17,360 (2.6%)	1,038 (6.0%)		0.90 (0.84, 0.97)	0.71 (0.65, 0.76)
Hispanic[a]	Female	18,272 (2.7%)	1,238 (6.8%)		1.08 (1.00, 1.17)	0.90 (0.84, 0.96)
	Male	23,469 (3.5%)	1,343 (5.7%)		0.84 (0.78, 0.90)	0.77 (0.72, 0.82)
White	Female	174,221 (25.9%)	15,758 (9.0%)		1.33 (1.29, 1.37)	1.21 (1.17, 1.24)
	Male	294,880 (43.8%)	21,372 (7.2%)		Reference	Reference
Other[b]	Female	7,625 (1.1%)	522 (6.8%)		0.57 (0.45, 0.73)	1.13 (1.04, 1.24)
	Male	8,729 (1.3%)	468 (5.4%)		0.43 (0.34, 0.54)	0.94 (0.85, 1.03)

* HR=Hazard Ratio
[a] Hispanic, Latino, of Spanish Origin, or Multi-Race Hispanic
[b] Other, Multi-Race Non-Hispanic, or Unknown

- 673,573 graduates were included in the analysis. 52,058 graduates were appointed to instructor (events). 621,515 graduates were censored: 507,369 were never appointed to instructor and were censored on the last date of file or maximum allowed time (7,539 days) whichever was earlier; 113,409 were appointed to a rank higher than instructor and were censored on first day of higher appointment; and 737 were later appointed to instructor but censored because they reached the maximum allowed time before instructor appointment (7,539 days).

© 2024 Clark L et al. *JAMA Network Open.*

eTable 2. Graduated Medical School to Assistant Professor

Race	Gender				Graduation Date	
					**Before 2000**	**After 2000**
				**N (Row %)**	330,396 (49.1%)	343,177 (51.0%)
				**Events (% of N)**	63,290 (19.2%)	63,130 (18.4%)
		**N (Column %)**	**Events (% of N)**	**HR* (95% CI)**		
**American Indian, Alaska Native, Native Hawaiian, or Pacific Islander**	**Female**	1,260 (0.2%)	166 (13.2%)		0.65 (0.54, 0.78)	0.56 (0.47, 0.67)
	**Male**	1,620 (0.2%)	230 (14.2%)		0.71 (0.60, 0.83)	0.60 (0.51, 0.71)
**Asian**	**Female**	46,326 (6.9%)	9,222 (19.9%)		1.23 (1.19, 1.27)	1.11 (1.08, 1.14)
	**Male**	55,915 (8.3%)	10,345 (18.5%)		1.09 (1.06, 1.12)	0.97 (0.95, 1.00)
**Black**	**Female**	23,896 (3.6%)	4,068 (17.0%)		0.98 (0.94, 1.02)	0.84 (0.80, 0.87)
	**Male**	17,360 (2.6%)	2,586 (14.9%)		0.84 (0.80, 0.88)	0.71 (0.68, 0.75)
**Hispanic[a]**	**Female**	18,272 (2.7%)	3,071 (16.8%)		0.96 (0.91, 1.01)	0.97 (0.93, 1.02)
	**Male**	23,469 (3.5%)	3,461 (14.7%)		0.82 (0.78, 0.85)	0.82 (0.79, 0.86)
**White**	**Female**	174,221 (25.9%)	36,239 (20.8%)		1.14 (1.12, 1.16)	1.15 (1.13, 1.17)
	**Male**	294,880 (43.8%)	54,958 (18.6%)		Reference	Reference
**Other[b]**	**Female**	7,625 (1.1%)	1,064 (14.0%)		0.51 (0.44, 0.59)	1.06 (1.00, 1.13)
	**Male**	8,729 (1.3%)	1,010 (11.6%)		0.44 (0.38, 0.51)	0.91 (0.86, 0.98)

* HR=Hazard Ratio

[a] Hispanic, Latino, of Spanish Origin, or Multi-Race Hispanic

[b] Other, Multi-Race Non-Hispanic, or Unknown

- 673,573 graduates were included in the analysis. 126,420 graduates were appointed to assistant professor (events). 547,153 graduates were censored: 534,600 were never appointed to assistant professor and were censored on the last date of file or maximum allowed time (7,539 days) whichever was earlier; 7,142 were appointed to a rank higher than assistant professor and were censored on first day of higher appointment; and 5,411 were later appointed to assistant professor but were censored because they reached the maximum allowed time before assistant professor appointment (7,539 days).

© 2024 Clark L et al. *JAMA Network Open.*

eTable 3. Instructor to Assistant Professor

Race	Gender				Graduation Date	
					Before 2000	After 2000
				N (Row %)	25,134 (48.4%)	26,770 (51.58)
				Events (% of N)	12,479 (49.6%)	11,747 (43.9%)
		N (Column %)	Events (% of N)	HR* (95% CI)		
American Indian, Alaska Native, Native Hawaiian, or Pacific Islander	Female	64 (0.1%)	31 (48.4%)		1.14 (0.75, 1.74)	0.80 (0.52, 1.24)
	Male	71 (0.1%)	35 (49.3%)		1.34 (0.91, 1.95)	0.82 (0.53, 1.27)
Asian	Female	4,074 (7.8%)	1,846 (45.3%)		0.98 (0.91, 1.05)	0.93 (0.87, 0.99)
	Male	4,275 (8.2%)	1,950 (45.6%)		1.09 (1.02, 1.16)	0.90 (0.85, 0.96)
Black	Female	1,749 (3.4%)	742 (42.2%)		0.72 (0.65, 0.79)	0.88 (0.80, 0.97)
	Male	1,043 (2.0%)	469 (45.0%)		0.83 (0.75, 0.92)	0.89 (0.79, 1.00)
Hispanic[a]	Female	1,244 (2.4%)	522 (42.0%)		0.74 (0.66, 0.84)	0.90 (0.81, 0.99)
	Male	1,341 (2.6%)	557 (41.5%)		0.81 (0.73, 0.90)	0.85 (0.76, 0.95)
White	Female	15,703 (30.3%)	7,447 (47.4%)		0.86 (0.83, 0.90)	0.99 (0.95, 1.03)
	Male	21,354 (41.1%)	10,228 (47.9%)		Reference	Reference
Other[b]	Female	520 (1.0%)	204 (39.2%)		1.00 (0.72, 1.39)	0.94 (0.82, 1.09)
	Male	466 (0.9%)	194 (41.6%)		1.21 (0.89, 1.66)	1.00 (0.86, 1.16)

* HR=Hazard Ratio

[a] Hispanic, Latino, of Spanish Origin, or Multi-Race Hispanic

[b] Other, Multi-Race Non-Hispanic, or Unknown

- Of the original sample of 673,573, 620,778 individuals were excluded because they never held an instructor appointment, and 891 individuals were excluded because they were appointed to assistant professor before being appointed to instructor. 51,904 Instructors were included in the analysis. 24,226 instructors were appointed to assistant professor (events). 27,678 instructors were censored: 52 were later appointed to assistant professor but were censored because they reached the maximum allowed time before assistant professor appointment (7,538 days), 5,078 were censored on the last date of file, and 22,548 were censored on last date of current instructor appointment plus 3 years

© 2024 Clark L et al. *JAMA Network Open.*

eTable 4. Summary of Appointments and Promotions

This table draws from data displayed in Tables 1-3 and eTables 1-3. In this table the point estimate of each hazard ratio is displayed with rows for each race/ethnicity and gender category and promotion levels in the columns. Numbers in bold indicate lower likelihood of promotion or appointment than White men. Numbers in italic font indicate higher likelihood of promotion than White men. Grey numbers signify cells where the CI for that gender and race/ethnicity contains 1 and thus are no different than White men.

Race	Gender	Graduated to Instructor		Graduated to Assistant		Instructor to Assistant		Assistant to Associate		Associate to Full		Enter Academia to Chair	
		Before 2000	After 2000	Before 2000	After 2000	Before 2000	After 2000	Before 2000	After 2000	Before 2000	After 2000	Before 2000	After 2000
American Indian, Alaska Native, Native Hawaiian, or Pacific Islander	Female	**0.69**	**0.59**	**0.65**	**0.56**	1.14	0.80	0.87	0.88	0.61	0.46	0.38	0
	Male	**0.57**	**0.53**	**0.71**	**0.60**	1.34	0.82	**0.68**	**0.70**	0.90	0.86	0.26	0
Asian	Female	*1.37*	*1.21*	*1.23*	*1.11*	0.98	**0.93**	**0.79**	**0.71**	1.05	0.85	**0.26**	**0.39**
	Male	*1.07*	1.04	*1.09*	0.97	*1.09*	**0.90**	*1.15*	1.04	*1.16*	*1.21*	0.88	1.13
Black	Female	*1.20*	**0.85**	0.98	**0.84**	**0.72**	**0.88**	**0.45**	**0.51**	**0.59**	**0.53**	**0.48**	1.20
	Male	**0.90**	**0.71**	**0.84**	**0.71**	**0.83**	0.89	**0.58**	**0.67**	**0.82**	0.94	*1.29*	*2.72*
Hispanic[a]	Female	1.08	**0.90**	0.96	0.97	**0.74**	**0.90**	**0.56**	**0.55**	**0.78**	**0.62**	**0.48**	1.12
	Male	**0.84**	**0.77**	**0.82**	**0.82**	**0.81**	**0.85**	**0.73**	**0.72**	**0.85**	0.87	0.92	*1.83*
White	Female	*1.33*	*1.21*	*1.14*	*1.15*	**0.86**	0.99	**0.77**	**0.77**	**0.85**	**0.66**	**0.40**	**0.48**
	Male	Reference	Reference	Reference	Reference	Reference	Reference	Reference	Reference	Reference	Reference	Reference	Reference
Other[b]	Female	**0.57**	*1.13*	**0.51**	1.06	1.00	0.94	**0.51**	**0.57**	0.99	0.56	1.26	0.70
	Male	**0.43**	0.94	**0.44**	**0.91**	1.21	1.00	0.86	0.96	0.91	0.66	1.08	0.51

[a] Hispanic, Latino, of Spanish Origin, or Multi-Race Hispanic
[b] Other, Multi-Race Non-Hispanic, or Unknown

© 2024 Clark L et al. *JAMA Network Open.*

eFigure. Summary of Appointments and Promotions Before and After 2000

eFigure, A: Summary of Appointments and Promotions – Before 2000

eFigure A and B compare promotions and appointments between cohorts before and after the year 2000. eFigure A. summarizes cohorts who graduated prior to 2000 with rows for each promotion level and columns for each race/ethnicity and gender category; eFigure B similarly summarizes cohorts graduating after 2000. Cells in red indicate lower likelihood of promotion or appointment than White men, while cells in dark blue indicate higher likelihood of promotion or appointment compared to White men. White cells correspond to race/ethnicity and gender groups where the confidence interval of the likelihood of promotion or appointment contains 1 and thus are no different than White men.

Race	American Indian, Alaska Native, Native Hawaiian, or Pacific Islander		Asian		Black		Hispanic[a]		White		Other [b]	
**Gender**	**Male**	**Female**	**Male**	**Female**	**Male**	**Female**	**Male**	**Female**	**Male**	**Female**	**Male**	**Female**
**Graduated to Instructor**									Reference			
**Graduated to Assistant**									Reference			
**Instructor to Assistant**									Reference			
**Assistant to Associate**									Reference			
**Associate to Full**									Reference			
**Enter Academia to Chair**									Reference			

eFigure, B: Summary of Appointments and Promotions – After 2000

Race	American Indian, Alaska Native, Native Hawaiian, or Pacific Islander		Asian		Black		Hispanic[a]		White		Other[b]	
**Gender**	**Male**	**Female**	**Male**	**Female**	**Male**	**Female**	**Male**	**Female**	**Male**	**Female**	**Male**	**Female**
**Graduated to Instructor**									Reference			
**Graduated to Assistant**									Reference			
**Instructor to Assistant**									Reference			
**Assistant to Associate**									Reference			
**Associate to Full**									Reference			
**Enter Academia to Chair**									Reference			

© 2024 Clark L et al. *JAMA Network Open.*

[a] Hispanic, Latino, of Spanish Origin, or Multi-Race Hispanic
[b] Other, Multi-Race Non-Hispanic, or Unknown

© 2024 Clark L et al. *JAMA Network Open.*
