# Supplementary material for: Race and Ethnicity, Gender, and Promotion of Physicians in Academic Medicine
Source: JAMA Netw Open. 2024 Nov 27;7(11):e2446018. doi: 10.1001/jamanetworkopen.2024.46018 (PMC12262150; doi:10.1001/jamanetworkopen.2024.46018)
Supplement: Supplement 2. — Data Sharing Statement [file jamanetwopen-e2446018-s002.pdf]

Data Sharing Statement

Clark. Race and Ethnicity, Gender, and Promotion of Physicians in Academic Medicine. *JAMA Netw Open*. Published November 27, 2024. doi:10.1001/jamanetworkopen.2024.46018

Data
Data available: No

Additional Information
Explanation for why data not available: Data is available from the Association of American Medical Colleges ("AAMC") through their data sharing request process.
